# Long-Term Outcomes of Patients with Digestive Tract Congenital Anomalies and Their Caregivers in Uganda: A Cohort Study

**DOI:** 10.1177/30502225251330496

**Published:** 2025-04-15

**Authors:** Nasser Kakembo, Ava Yap, Ann Nabirye, Phyllis Kisa, Stella Nimanya, Innocent Okello, Rovine Naluyimbazi, Fiona Mbwali, Peter Kayima, Yasin Ssewanyana, John Sekabira, Doruk Ozgediz

**Affiliations:** 1 Makerere University College of Health and Sciences, Kampala, Uganda; 2 University of California San Francisco, San Francisco, CA, USA; 3 Mulago Hospital, Kampala, Uganda

**Keywords:** low-income countries, congenital anomalies, mortality risk, long-term outcomes, caregiver burden, quality of life

## Abstract

In Uganda, long-term outcomes remain unknown for children living with digestive tract congenital anomalies (DTCA) after corrective surgery or hospitalization. This cohort study set in a tertiary hospital in Uganda investigated the long-term outcomes of children with DTCAs. Caregivers with deceased children completed a verbal autopsy, while those with live children completed a PedsQL quality of life (QoL) survey. Multivariate Cox proportional hazard regression and linear regression explored factors associated with death and quality of life, respectively. Of the 362 participating caregivers, 265 (73.2%) had a child with a DTCA who survived since hospital discharge. The median age and follow-up time were 3.2 years. The mortality hazard ratio for children with DTCAs was 2.70 (95% confidence interval 2.21-3.30). Mortality was associated with DTCA diagnosis, the presence of stoma, and higher household income. Our findings support the urgent need for outpatient community healthcare services specific to DTCA patients to counteract these consequences.

## Introduction

Congenital anomalies account for 6% of births and 300,000 newborn deaths yearly, making it a leading cause of neonatal and child death.^1^ Ranked as the fifth highest contributor of disability worldwide, the disease burden of congenital anomalies is predominantly shouldered by low- and middle-income countries (LMIC).^2^ Definitive repair of many congenital anomalies (CAs) requires surgery, as recognized by the World Health Organization (WHO).^1^ However, an estimated 1.7 billion children worldwide lack access to surgical care, with the majority residing in LMIC where the proportion with no access reaches 92% to 98%.^3^ This scarcity leads to dire consequences in babies born with surgically correctable diseases. A prospective cohort study spanning 19 Sub- Saharan African (SSA) countries demonstrated that neonatal and infant mortality from digestive tract congenital anomalies (DTCAs) remains markedly higher than that of high-income countries (HICs) for gastroschisis (75.5% vs 2.0%) and anorectal malformations (11.2% vs 2.9%), despite preexisting pediatric surgical services in SSA.^4^ Delays in the management of congenital anomalies are also common and lead to preventable disease burden. In a review of 5 common congenital anomalies in 13 African countries, delay time to surgery averaged 2 years and surgical backlog ranged from 6299 cases for cleft lip to 320 777 for cryptorchidism.^5^ Inability to timely treat these malformations accumulated ~75 000 otherwise preventable disability-adjusted life years (DALYs).^5^

At Uganda’s national referral hospital, patients with DTCAs can be definitively treated with surgery by one of the few pediatric surgical teams in the country. However, many patients continue to have persistent symptoms and disability that are not addressed such as ostomy care, chronic fecal incontinence, or gastrointestinal dysmotility.^6^ These issues can lead to complications that hinder the child’s quality of life and development if left untreated, and no consistent follow-up care is provided to ensure optimal recovery after a surgical admission.^7^ Furthermore, long-term survival is unknown in this patient population.

Community health extension workers (CHEWs) could serve as the healthcare liaisons needed by children living with CAs to monitor and address these issues in a timely fashion. The WHO and UNICEF recognize that facility-based services alone are often insufficient to provide prompt and equitable care, especially in LMICs, and that CHEWs are a viable alternative.^8^ CHEWs are deployed in 16 SSA countries and have demonstrated improved survival for chronic childhood conditions in LMICs including malaria, diarrhea, and pneumonia, though none have been described for surgically correctable CA.^9^

Therefore, finding a suitable outreach strategy to care for these patients outside the hospital is necessary, and to do so requires a baseline understanding of factors affecting morbidity and mortality in children with a documented DTcA diagnosis in Uganda. The overarching goals of this study were to (1) investigate the long-term outcomes of children treated with DTCAs and (2) perform a needs assessment for families caring for these children in Uganda to inform a pilot outreach program.

## Methods

### Overall Study Design

This was a prospective cohort study of children with DTCAs who were discharged alive from the pediatric surgery service in Uganda’s national referral hospital from February 2015 to June 2022. Ethical approval was obtained through the Institutional Review Boards (IRBs) of Makerere University School of Medicine (#2022-354) and the University of California San Francisco (#22-36957).

### Data Source and Participants

Participants were caregivers of pediatric patients from 0 to 17 years old with a confirmed diagnosis of a DTCA, with or without corrective surgery. These individuals must have had at least 1 documented admission to the inpatient pediatric surgical service. Diagnoses of included DTCAs are displayed in Table 1. Exclusion criteria were patients who passed away during their hospital stay, patients who were less than 6 months out from their initial contact with the hospital’s pediatric surgical service, caregivers who did not have means of remaining in contact via phone, and individuals who did not speak English or Luganda. The 6-month waiting period was chosen to allow time for long-term outcomes to manifest.

**Table 1.** Diagnoses of Digestive Tract Anomalies Included in Our Study Cohort.

Category	Diagnosis
Abdominal wall defects	Omphalocele
	Gastroschisis
Colorectal anomalies	Anorectal malformation
	Hirschsprung’s disease
	Cloacal malformation
Small intestinal anomalies	Intestinal atresia
	Malrotation
Esophageal	Tracheoesophageal fistula

Potential participants were identified using the inpatient and outpatient pediatric surgical ward registry, an electronic database holding records of patients with DTCAs since 2012 when the database was conceived. Consecutive sampling was undertaken, starting with the most recent recorded admission and working backward chronologically. In this way, we reached those who were the youngest first or had the most recent documented visits with our ward, which optimized the chances of successful contact. The local research team determined participant eligibility for recruitment based on the prespecified list of DTcAs that were treated by the pediatric surgical service. Potential participants were contacted via phone by a local research assistant fluent in English and Luganda. Participants provided verbal consent, which was approved by the respective IRBs given the virtual nature of the data collection method and the minimal risk the study posed to subjects.

To establish a comparison group, childhood mortality data were obtained from the 2016 Uganda Demographic and Health Survey (DHS), which was a nationwide survey conducted over a representative sample of 19 588 households across 696 community clusters.^10^ Participant-level data included the vital status of the pediatric population and age at the time of death. Children 17 years old or younger who were born to mothers aged 15 to 49 years old in the DHS 2016 were included in this study as the comparison group.

### Instruments

The phone-based survey assessed the caregivers’ and pediatric surgical patients’ demographics, current functional status, perioperative characteristics, and caregiver burden as a result of the surgery. Survey instruments were derived from pre-existing tools, which were validated in similar settings and piloted in a small subset of participants for comprehension and ease of administration. The research assistant conducting the survey entered responses electronically into a secure REDCap platform using a tablet.

Caregivers who reported that their child had died participated in an abbreviated version of the World Health Organization (WHO) Verbal Autopsy (VA), which documented the child’s cause of death to determine if the death was related to the child’s DTCA. The VA is a validated tool used to improve documentation of vital statistics in LMIC settings and its use has been well described in prior Ugandan studies, including a 2007 Child Verbal Autopsy Study conducted by the Uganda Bureau of Statistics.^11-13^ In Uganda, community health workers successfully administered the VA to marginalized, rural communities to determine causes of under-5 mortality.^14,15^ In our study population, questions derived from the VA evaluated the circumstances surrounding the child’s death, including the age at the time of death, the probable cause of death, and the events and symptoms leading up to death.

For caregivers who reported that their child was alive at the time of the survey, the Pediatric Quality of Life Inventory 4.0™ (PedsQL™) ascertained the child’s quality of life (QoL) across 4 main domains: physical functioning, emotional/cognitive functioning, social integration, and school performance in school-age children.^16,17^ This instrument is one of the most widely utilized child-specific QoL measurement tools internationally, is available in the Luganda language and is validated in the Ugandan pediatric population. It has been used in prior studies in Ugandan children suffering from other chronic illnesses such as rheumatic heart disease, HIV, and chronic kidney disease.^18-20^ The PedsQL score is scaled from 0 to 100 with a score of <80 indicating a significant reduction in QoL based on prior studies.

### Variables of Interest

Our primary predictor was the child’s DTCA diagnosis, as listed in Table 1. Our primary outcome of interest was the vital status of the child at the time of the survey. For those alive, a secondary outcome was the child’s quality of life, determined by the PedsQL score. For those deceased, a secondary outcome was long-term cumulative mortality, which was the combined mortality of inpatients and outpatients documented in the ward database. This cumulative mortality was compared to the average 30-day mortality reported in a prospective cohort study of 9 low-income countries (LICs).^21^

Additionally, the caregiver’s belief of their child’s DTCA contributing to their death was recorded, characterized by the qualifiers: unlikely, possibly, or likely related. We assessed the concordance between caregiver responses and the clinician’s assessment of the death relation to the child’s DTCA condition. The clinician’s assessment was based on 2 physicians’ review of the verbal autopsy reports. Covariates included patient age, sex, caregiver relationship to the child, residential district, religious affiliation, education level of the caregiver, household makeup, and caregiver marital and socioeconomic status. Residential districts were categorized into 3 groups, those surrounding the hospital (ie, Wakiso, Kampala, and Mukono), those in the greater Ugandan region, and those in other regions.

### Statistical Analysis

Outcomes were analyzed with descriptive statistics and tests of association using Stata 16.0 MP (College Station, TX). A *P*-value of less than <.05 was considered statistically significant. Test of differences between groups utilized a chi-squared test for categorical variables (e.g. biological sex), Student’s *t*-test for normally distributed continuous variables (e.g. age), and Mann-Whitney *U* test for continuous variables with skewed distribution (e.g. family income). To evaluate the caregiver’s understanding of the cause of death, a kappa statistic with linear weights measured the interrater reliability between the caregiver and physician on the assessment of the child’s death’s relation with the DTCA.

VA responses that were missing age at the time of death (n = 21) were imputed using a truncated regression imputation method.^22^ This method is used to address missing values of a continuous variable with a finite range, such as patients’ age. This imputation provided an asymptotic approximation of missing values of age using the variable’s posterior predictive distribution. A right-censored Kaplan Meier analysis estimated the survival distribution of our sample population, using the date of birth as the study start time. A log-rank test compared the survival time of our patient population with that of the general pediatric population and among disease categories, using the child mortality rates reported in the 2016 Uganda DHS as our reference. Univariate and multivariate Cox proportional hazards regression models with listwise deletion predicted the risk of death among diagnosis categories and associations with patient factors. Model covariates were chosen based on their potential as confounders and differences in prevalence between the deceased and live groups with a *P* < .1 (Table S1).

Subgroup analysis was performed to assess factors associated with a higher quality of life in children who were alive. A multivariate linear regression model with listwise deletion was performed to evaluate independent predictors of a higher PedsQL score.

## Results

In total, 362 caregivers participated in the phone survey. The median age of caregivers was 34 years old (range: 18-72 years old), and most caregiver respondents were birth mothers (64.3%, n = 218), Christian (61.9%, n = 211), and married (83.8%, n = 285), though 12.1% (n = 41) of families were divorced or separated. Approximately half (48.8%, n = 166) of the families reside in the nearby districts and had a mean annual household income of 830 U.S. dollars ($). The median age of the child was 3.2 years old, and 76.5% (n = 261) underwent operative repair. Anorectal malformations were the most common diagnosis group (41%, n = 150). As a result of surgery, 10.7% (27) families were divided, 7.0% (n = 18) of caregivers lost their job, 11.5% (n = 29) went into debt, and 65% (n = 98) suffered catastrophic healthcare expenditure due to their child’s surgery.

Since hospital discharge, 26.8% (n = 97) of the children with DTCAs had died, of which 21.6% (n = 21) did not complete the full VA. Compared to caregivers with children who had died, those with children who were alive were more likely to earn a lower household income ($1116 vs $765, *P* = .038), be younger (34 years old vs 37 years old, *P* = .008), and be divorced or separated 14.0% versus 5.3%, *P* = .039; Table S1). The median age of the child’s death was 4.4 months (interquartile range [IQR] 1.1-17.1 months). Most children died in the hospital (54%, n = 41), and almost all caregivers thought their child’s death was unexpected (96%, n = 73). Symptoms started within a week prior in 70 (92%) children who died, while 35 (46%) developed symptoms within a day before their death. The most common symptoms were fever (33%, n = 25), difficulty feeding (29%, n = 22), and abdominal pain (28%, n = 21). The presence of symptoms prior to death is showcased in Figure S1 in the Supplemental Materials.

**Figure 1.**
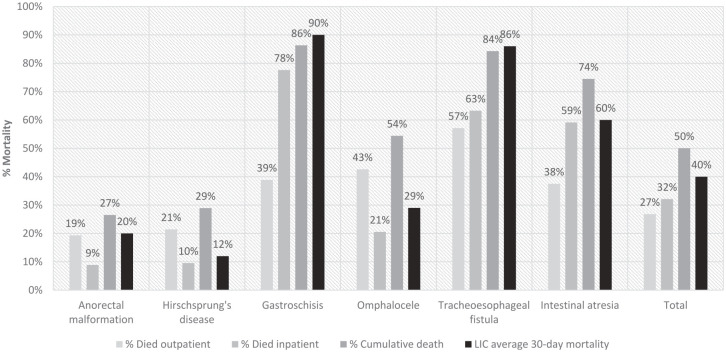
Inpatient, outpatient, and long-term cumulative mortality of our patient cohort for select abdominal congenital anomalies, compared to the average 30-day mortality reported in a previous prospective cohort study involving 9 low-income countries.^21^ The cumulative mortality of our patient population is comparable to higher than previously reported values in the literature, though our observation period is longer (median follow-up of 3.17 years).

**Figure 2.**
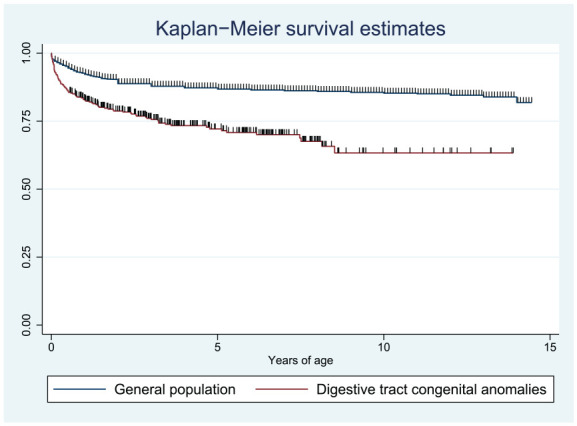
Kaplan-Meier survival curves of children with digestive tract congenital anomalies (DTCAs) within our study cohort compared to the general pediatric population. Compared to the general population, children with DTCAs were significantly less likely to survive (log-rank test *P* < .001).

The median follow-up time for children with DTCAs was 3.17 years (IQR 1.11-6.34), while the median follow-up time for the general pediatric population was 6.73 (IQR 2.1-10.6). The overall survival for the general pediatric population in the 2016 DHS was 87.0% (41 478/47 701), while the overall survival for the DTCA population was 73.2% (265/362). When including those who died as inpatients, the cumulative mortality of our patient cohort was 50.0%. The cumulative mortality of select DTCAs is showcased in Figure 1, with the average 30-day mortality from a prior prospective cohort study of 9 LICs as a reference. Outpatient mortality by congenital anomaly diagnosis is shown in Supplemental Table S2. Of those who provided the child’s age at the time of death, 67.1% (51/76) died within the first year of life. In our survival analysis, children with DTCAs had a shorter survival time than the general pediatric population (log-rank test *P* < .001), or 2.70 times the hazard for death (95% confidence interval [CI] 2.21-3.30; Figure 2).

**Figure 3.**
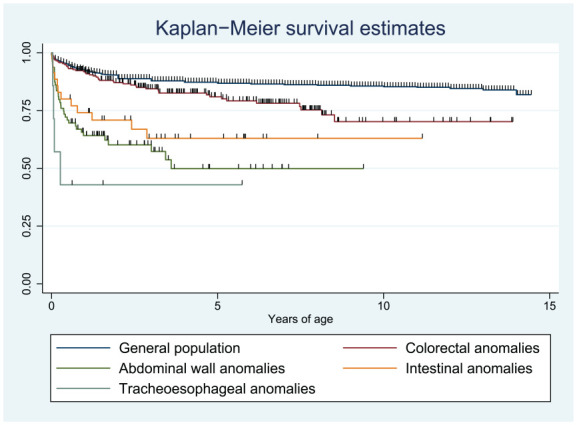
Kaplan-Meier survival curves of children subdivided over digestive tract congenital anomaly (DTCA) groups, compared to the general pediatric population. Among children with DTCAs, tracheoesophageal anomalies had the lowest survival, while colorectal anomalies had the best survival.

Children with colorectal anomalies were more likely to survive longer than those with abdominal wall defects, intestinal anomalies, and tracheoesophageal fistulas (log-rank test *P* < .001; Figure 3). Compared to the general pediatric population, the unadjusted mortality hazard ratio (HR) was 1.82 (95% CI 1.37-2.42) in children with colorectal anomalies, 5.40 for children with abdominal wall defects (95% CI 3.85-7.63), 3.81 (95% CI 2.16-6.72) for children with intestinal anomalies, and 10.9 (95% CI 4.08-29.0) for children with tracheoesophageal fistulas.

A multivariate Cox proportional regression including 216 respondents was performed to model mortality risk, controlling for sex, diagnosis category with colorectal anomalies as the reference, presence of stoma, the residential region, household income, and divorced caregivers. In this model, the diagnosis category (specifically abdominal wall defects, intestinal anomalies, and tracheoesophageal fistulas), the presence of a stoma, and higher household income were found to be independently associated with mortality (Table 2).

**Table 2.** Hazard ratios of the Multivariate Cox Proportional Regression of Mortality Hazard Associated with the Study Cohort’s Patient and Caregiver Factors.

Variables (n = 216, deaths = 39)	Hazard ratio	95% CI	*P*-value
Sex	0.896	(0.452-1.779)	.754
Colorectal anomalies (reference)	1		
Abdominal wall defects	6.949	(2.440-19.79)	<.001
Intestinal anomalies	3.721	(1.020-13.58)	.047
Tracheoesophageal fistula	39.14	(8.750-175.1)	<.001
Presence of stoma	5.288	(1.885-14.83)	.002
Kampala/Wakiso/Mukono (reference)	1		
Greater central region	1.500	(0.599-3.751)	.386
Other regions	1.000	(0.470-2.131)	.999
Annual income (per $100)	1.03	(1.008-1.052)	.007
Caregivers divorced or separated	1.178	(0.340-4.080)	.796

Of the caregivers who completed the VA, 29 (38%) believed that the cause of death was likely related to the child’s CA, 38 (50%) believed it was possibly related, and 9 (12%) believed it was unlikely related. In the physician’s review of the VA, 31 (41%) of deaths were likely related to the child’s CA, while 34 (45%) were possibly related, and 11 (14%) were unlikely to be related. Agreement between caregiver and physician was 85.5%. The kappa statistic between the caregiver and physician’s assessment of the cause of the child’s death in relation to the DTCA was 0.591 (*P* < .001), demonstrating moderate interrater reliability.

Children who were alive (N = 265) had a median age of 4.2 years old (IQR 2.6-6.9) and 50.2% (113) came from nearby districts. Of those with live children, 264 (99.6%) participants provided PedsQL responses. The mean overall PedsQL score was 89.68 (SD 10.04). The overall physical summary score was 84.57 (SD 12.67) and the overall psychosocial summary score was 93.29 (11.08). The overall PedsQL score was not significantly different across DTCA diagnoses (*P* = .068), but the PedsQL psychosocial summary score was significantly lower in patients with tracheoesophageal atresias (80.35 vs 88.17-95.74, *P* < .001; Table S3). Notably, patients with stomas tended to have lower PedsQL scores in every single domain, with the difference most pronounced in the physical domain (Table 3). In a multivariate logistic regression model controlling for diagnosis, sex, age, residential region, caregiver unemployment, presence of caregiver debt, and child school attendance, a higher PedsQL score was associated with the absence of a stoma (β = 3.8, 95% CI 0.5-7.0), increasing age (β = .6 for each additional year, 95% CI 0.1-1.0), and attending school (β = 6.3, 95% CI 2.8-9.7).

**Table 3.** PedsQL Scores for Patients who were Alive, Subdivided by Those who did or did not have Stomas. Note that Schooling Performance is not Included in this Table Given this Domain is not Applicable to Most Children with Stomas, Who do not Attend School.

	Total	No stoma	With stoma	
PedsQL domain	N = 263	N = 247	N = 93	*P*-value
PedsQL physical health domain	83.30 (13.98)	85.16 (12.37)	77.86 (16.83)	<.001
PedsQL emotional health domain	95.85 (8.84)	97.03 (6.55)	92.40 (12.93)	<.001
PedsQL social functioning domain	91.29 (15.29)	93.37 (14.35)	85.21 (16.41)	<.001
PedsQL physical health summary	84.57 (12.67)	86.44 (10.39)	79.11 (16.67)	<.001
PedsQL psychosocial health summary	93.29 (11.08)	94.90 (10.06)	88.59 (12.59)	<.001
PedsQL total score	89.68 (10.04)	91.43 (8.63)	84.54 (11.99)	<.001

## Discussion

This cohort study investigated the long-term outcomes of children with DTCAs who were discharged from a national referral hospital in Uganda. To our knowledge, this is the first study to look at vital status and QoL in children with DTCA after receiving care from a pediatric surgery service in an LMIC. We found that over three quarters of the children survive years after their index surgery. Furthermore, most children who live have high QoL and little to no functional deficits. However, the all-cause, long-term mortality is still significantly higher than that of the general pediatric population, with most deaths possibly attributable to the DTCA condition. Most children die in the first year of life, providing the opportunity for a CHEW program to intervene during this high-risk period.

In our cohort of patients who were discharged from the hospital, 74% of children with DTCAs survived over a median of 3.2 years after discharge from the pediatric surgery service. When including both inpatients and outpatients in our study, the long-term cumulative survival fell to 50%, which was comparable to prior studies (Figure 1). Our findings are consistent with other studies in LMICs, which have shown similar disparity in survival compared to HIC patients, with survival differences most stark for gastroschisis (survival 24.5% in LMICs vs 98.0% in HICs) and anorectal malformations (survival 88.8% in LMICs vs 97.1% in HICs).^4^ While long-term survival has not been previously reported in this patient population, 30-day and inpatient survival rates of pediatric surgical patients in LMICs have been studied. A previous study reported inpatient survival for children with CAs at 74%,^23^ with the inpatient survival for neonates was lower at 64%.^24^ Newborns with intestinal atresias commonly present a week late and therefore suffer a preventable 43% mortality rate due to sepsis, malnutrition, and complications from associated anomalies in-hospital.^25^ The average 30-day survival was 60% after a primary intervention for children with DTCAs in 9 low-income countries.^21^ For abdominal surgical emergencies, 30-day survival was 76%, 91%, and 96% for neonates, infants, and older children, respectively.^26^ In the general pediatric surgical population, in-hospital survival was 58.1% for neonates, 95% for infants, and 99% for older children in a prospective cohort study involving 19 SSA countries.^4^ Our comparatively lower long-term survival is expected, as we captured additional mortality events over years. Nevertheless, half of the patients in our cohort preserved long-term survival, which is a testament to the dedicated delivery of surgical care by the pediatric surgical service. Outcomes in conditions that were previously uniformly fatal were substantially improved by life-saving surgery.

Notably, Ugandan children with DTCAs who were discharged from their index admission still had a mortality hazard that was 2.4 times higher than the general pediatric population. Among DTCA diagnoses, the mortality hazard climbs further for abdominal wall defects and tracheoesophageal fistulas. High rates of mortality in these conditions corroborated findings from previous short-term studies and demonstrated the disparities in outcomes between HICs and LMICs.^21^ Gastroschisis had the widest gap in 30-day mortality at 90% in LICs versus 1.4% in HICs, and esophageal atresias had a similarly wide mortality gap at 86% in LICs versus 7.2% in HICs.^21^ Our disease-specific mortality were similar to prior studies in Uganda, where the mortality for jejunal atresia was 47% and gastroschisis neared 100%.^25,27^ Notably, our disease-specific mortality was not risk-adjusted, and previous studies have shown that delays in presentation exacerbate disease severity, with the adverse effects persisting after surgery and even discharge.

Of note, this mortality hazard appears to persist over time, even after surgery and successful discharge from the hospital, suggesting the need for outpatient monitoring for these patients. Two-thirds of the deaths in our patient population occurred within the first year. This finding is not surprising, considering that the 30-day mortality in neonates with pediatric surgical disease was significantly higher than that in older children.^4,26^ The general pediatric population reflects a similar predominance of deaths in very young children.

Up to 85% of the deaths may be related to the patients’ DTCAs based on a review of their VAs. Moreover, caregivers’ and physicians’ assessment of the cause of death appeared to have moderate concordance, suggesting that caregivers may be able to recognize when their child’s condition is deteriorating due to their DTCA and appropriately seek medical attention. This was substantiated by the fact that a large proportion of them sought medical treatment prior to death. However, there were several accounts of children perishing in transit before they are able to reach the hospital, a consequence in care delays have been previously describe.^28^ Therefore, improving outcomes of children with DTCAs within the first year of life holds the most potential impact, and could be the focus of a community outreach program.

In the patients who remained alive, both domain-specific and total PedsQL scores were generally above the 80-point threshold, signifying a good QoL. These findings are encouraging and suggest that patients who fully recover from definitive surgical repair go on to leave full, fulfilling lives with little functional decapacitation. However, stoma patients notably tended to have lower QoL scores in every single domain, most pronounced in the physical domains. In fact, children with stomas were more likely to not attend school and have families that went into debt because of their condition. This is not surprising, as previous studies have found that stoma care in Uganda engenders significant caregiver burden, chronic physical disability from stoma complications, and developmental delays due to social isolation.^6,29^ Specifically, stoma-associated prolapse and dermatitis can be chronically disabling, especially with no reliable stoma appliances that are affordable and available to most families.^30^ Therefore, it is important for the healthcare system to address these impairments and provide education and support in a sustained manner.

A CHEW program can incorporate elements of stoma teaching and care to better equip caregivers to manage these children’s specialized needs. While a DTCA-specific CHEW program does not currently exist in Uganda, multiple studies and organizations have substantiated CHEWs’ effectiveness in low-resource settings. The World Health Organization (WHO) and UNICEF highlight the potential of CHEWs as a viable alternative to hospital-centered care.^8^ CHEWs are currently active in 16 sub-Saharan African (SSA) countries, where they have contributed to improved survival rates for common high-burden childhood illnesses such as malaria, diarrhea, and pneumonia.^9^ To further support the potential for CHEWs to monitor DTCA patients, the WHO introduced a Training of Trainers program alongside a birth defect manual designed to enhance DTCA surveillance in 9 SSA countries.^31^ In Uganda, a community-based study on anorectal malformations found persistent bowel dysfunction in children years after corrective surgery.^6^ A pilot multidisciplinary bowel management program involving inpatient and community education and support for caregivers of DTCA patients showed promising results in reducing incontinence episodes and increasing caregiver confidence.^32^ This supporting evidence indicates that implementing a CHEW-led program in Uganda could be a valuable strategy for managing DTCAs and their long-term complications.

Pediatric surgery’s impact on family dynamics and economics was also notable, as 65% of families suffered catastrophic healthcare expenditure and 1 out of 10 families experienced separation or divorce. Other studies in Uganda have documented similar rates of catastrophic healthcare expenditure due to pediatric surgical disease, both in the inpatient and outpatient settings.^33,34^ Interestingly, families with higher income were more likely to have children who died (HR 1.03 for each additional $100 in annual income), suggesting that their economic burden lessened after the child had passed away. As such, the toll of caring for a child’s DTCA extends and has adverse effects on the QoL of the entire family unit. To delve into the nuances of caregiver burden, we are undertaking a qualitative study of caregiver focus groups to inform the design of the CHEW program that could address their specific unmet care needs.

Study strengths included the long duration over years in a patient population where long-term outcomes had not been previously studied. Furthermore, the prospective nature of the study allowed for causal inferences to be ascribed. Additionally, although single-center in nature, our study site is the national referral center of the country and receives Uganda’s most complex DTCA cases, such that our patient population represents the country’s severity of DTCA disease burden. Therefore, our findings might generalize to other LMICs in the region with a similar healthcare referral system.

Limitations of this study included its observational design and the self-reported survey format, which was reliant on respondent memory and subject to recall bias. This limitation specifically pertained to the ages reported at the time of death, which may have been subject to inaccuracy due to the caregiver report. Additionally, some respondents declined to complete the verbal autopsy so the missingness for the survival analysis was nonrandom and needed to be imputed. The mortality rates were also not risk-adjusted, such that patients with DTCA who presented to the hospital were likely already suffering the consequences of delayed presentation and inadequate resuscitation.

## Conclusion

This is the first study to investigate the long-term outcomes of children with DTCAs who were discharged from a pediatric surgical service in LMICs. We found that three-quarters of the children survive years after surviving their index admission, though the hazard of all-cause mortality remains more than twice as high as the general pediatric population. According to the VA report, most deaths were possibly related to the children’s DTCA. Conversely, children who survive maintain a high QoL in all domains, though those with stomas have significantly lower physical functioning. Despite inpatient treatment, children with DTCAs are still at risk of long-term morbidity and mortality after discharge, which substantiates the urgent need for a community outreach program to monitor and continue to support these patients. Extended community and outpatient follow-up is needed for children with DTCAs to sustain benefits from life-saving surgery at the beginning of life.

## Supplemental Material

sj-docx-1-gph-10.1177_30502225251330496 – Supplemental material for Long-Term Outcomes of Patients with Digestive Tract Congenital Anomalies and Their Caregivers in Uganda: A Cohort Study Supplemental material, sj-docx-1-gph-10.1177_30502225251330496 for Long-Term Outcomes of Patients with Digestive Tract Congenital Anomalies and Their Caregivers in Uganda: A Cohort Study by Nasser Kakembo, Ava Yap, Ann Nabirye, Phyllis Kisa, Stella Nimanya, Innocent Okello, Rovine Naluyimbazi, Fiona Mbwali, Peter Kayima, Yasin Ssewanyana, John Sekabira and Doruk Ozgediz in Sage Open Pediatrics

## Statements and Declarations

**Ethical Considerations:** Ethical approval for this study was obtained through the Makerere University School of Medicine (#2022-354) and the University of California San Francisco (#22-36957) Institutional Review Boards.

**Consent to Participate:** Participants’ verbal consent was obtained over phone. Through a two-way discussion, the research team member introduced their role, explained the purpose, components, risks, and benefits of the proposed study after given the permission to do so. Participants were able to ask clarifying questions or decline to participate in the study. Participants were assured that declining to participate would not affect their access to healthcare. Once understanding of the study was confirmed, verbal consent from the participant was obtained or participants could decline to participate. Verbal consent was documented using an online survey tool.

**Consent for Publication:** Consent for publication of the results of the study were obtained verbally. Research team members explained the purpose, components, risks, and benefits of the study’s publication and allowed the participants to decline study participation and publication.

**Data Availability:** This study’s registry data is not readily available as individual patient information may be sensitive and identifiable based on the rarity of some of the cases and patient diagnoses. Study data may be available from the corresponding author on select basis upon reasonable request.

**Supplemental Material:** Supplemental material for this article is available online.
