# Supplementary material for: Long-Term Outcomes of Patients with Digestive Tract Congenital Anomalies and Their Caregivers in Uganda: A Cohort Study
Source: Sage Open Pediatr. 2025 Apr 15;12:30502225251330496. doi: 10.1177/30502225251330496 (PMC12220909; doi:10.1177/30502225251330496)
Supplement: sj-docx-1-gph-10.1177_30502225251330496 – Supplemental material for Long-Term Outcomes of Patients with Digestive Tract Congenital Anomalies and Their Caregivers in Uganda: A Cohort Study [file sj-docx-1-gph-10.1177_30502225251330496.docx]

**Supplementary Materials**

Supplementary Figure S1: Bar graph showing the presence of symptoms before death for children who had a verbal autopsy conducted.


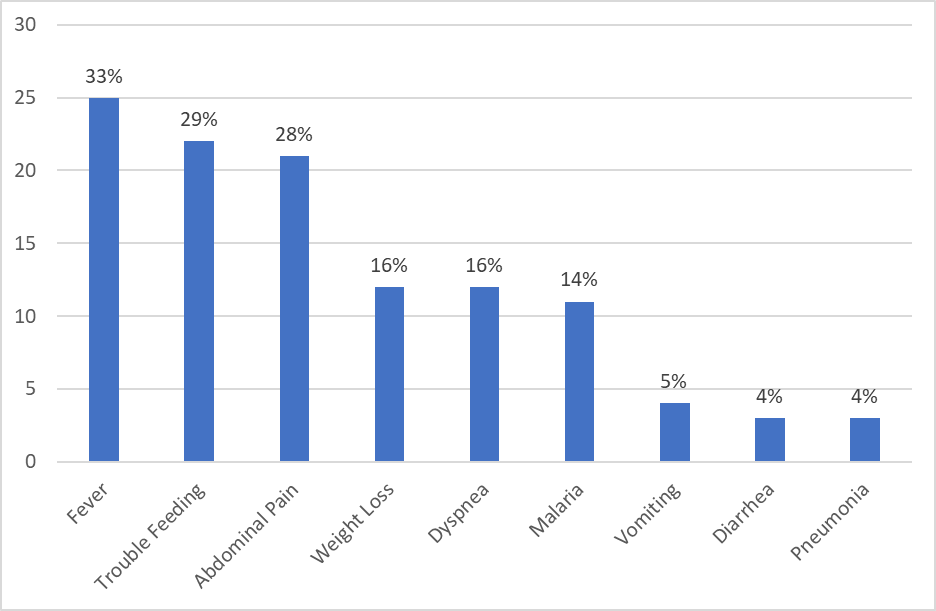


Supplementary Table S1: Baseline patient characteristics comparing those who were alive and dead. Continuous variables were compared using Student’s t-tests while categorical variables were compared using chi-squared tests.

|  | Total | Died | Alive | p-value |
| --- | --- | --- | --- | --- |
|  | N=362 | N=97 | N=265 |  |
| Age (at time of death or survey) | 3.17 (1.11-6.34) | 0.50 (0.11-1.88) | 4.21 (2.35-6.92) |  |
| Sex |  |  |  | 0.63 |
| Male | 181 (53.6%) | 42 (56.0%) | 139 (52.9%) |  |
| Female | 157 (46.4%) | 33 (44.0%) | 124 (47.1%) |  |
| Geographic region |  |  |  | 0.33 |
| Kampala/Wakiso/Mukono | 166 (48.8%) | 33 (44.0%) | 133 (50.2%) |  |
| Greater central region | 49 (14.4%) | 9 (12.0%) | 40 (15.1%) |  |
| Other regions | 125 (36.8%) | 33 (44.0%) | 92 (34.7%) |  |
| Diagnosis |  |  |  | 0.005 |
| Anorectal malformation | 150 (41.4%) | 29 (29.9%) | 121 (45.7%) |  |
| Hirschsprung's disease | 84 (23.2%) | 18 (18.6%) | 66 (24.9%) |  |
| Gastroschisis | 18 ( 5.0%) | 7 ( 7.2%) | 11 ( 4.2%) |  |
| Omphalocele | 61 (16.9%) | 26 (26.8%) | 35 (13.2%) |  |
| Tracheoesophageal fistula | 7 ( 1.9%) | 4 ( 4.1%) | 3 ( 1.1%) |  |
| Intestinal atresia | 32 ( 8.8%) | 12 (12.4%) | 20 ( 7.5%) |  |
| Cloaca | 4 ( 1.1%) | 1 ( 1.0%) | 3 ( 1.1%) |  |
| Cloacal exstrophy | 3 ( 0.8%) | 0 ( 0.0%) | 3 ( 1.1%) |  |
| Malrotation | 3 ( 0.8%) | 0 ( 0.0%) | 3 ( 1.1%) |  |
| OOP costs (USD) | 116.49 (123.39) | 104.19 (133.58) | 119.29 (121.14) | 0.46 |
| Annual income (USD) | 830.82 (977.11) | 1115.84 (1137.00) | 765.17 (927.68) | 0.038 |
| Respondent's relation to patient |  |  |  | 0.078 |
| Birth mother | 218 (64.3%) | 42 (55.3%) | 176 (66.9%) |  |
| Birth father | 112 (33.0%) | 33 (43.4%) | 79 (30.0%) |  |
| Another caregiver | 9 ( 2.7%) | 1 ( 1.3%) | 8 ( 3.0%) |  |
| Respondent's age | 34.43 (8.56) | 36.68 (8.88) | 33.78 (8.36) | 0.009 |
| Household religion |  |  |  | 0.008 |
| Christian | 211 (61.9%) | 56 (73.7%) | 155 (58.5%) |  |
| Muslim | 67 (19.6%) | 15 (19.7%) | 52 (19.6%) |  |
| Other | 63 (18.5%) | 5 ( 6.6%) | 58 (21.9%) |  |
| Divorced or Separated | 41 (12.1%) | 4 ( 5.3%) | 37 (14.0%) | 0.039 |
| Respondent education level |  |  |  | 0.76 |
| No formal education | 12 ( 3.5%) | 3 ( 3.9%) | 9 ( 3.4%) |  |
| Primary school | 104 (30.6%) | 26 (34.2%) | 78 (29.5%) |  |
| Secondary school | 160 (47.1%) | 32 (42.1%) | 128 (48.5%) |  |
| University | 59 (17.4%) | 13 (17.1%) | 46 (17.4%) |  |
| Advanced degree | 5 ( 1.5%) | 2 ( 2.6%) | 3 ( 1.1%) |  |
| Caregiver unemployed | 135 (39.7%) | 35 (46.7%) | 100 (37.7%) | 0.16 |
| # of members in the household | 5.90 (6.85) | 6.25 (9.46) | 5.80 (5.91) | 0.62 |
| # of children in the household | 3.75 (5.14) | 3.36 (1.83) | 3.85 (5.71) | 0.48 |
| Lives with spouse | 284 (85.0%) | 68 (90.7%) | 216 (83.4%) | 0.12 |
| stoma | 93 (27.4%) | 25 (32.9%) | 68 (25.8%) | 0.22 |

Supplementary Table S2: Outpatient mortality by abdominal congenital anomaly diagnosis

| Diagnosis | % Outpatient Mortality | # |
| --- | --- | --- |
| Anorectal malformation | 19% | (29/150) |
| Hirschsprung's disease | 21% | (18/84) |
| Gastroschisis | 39% | (7/18) |
| Omphalocele | 43% | (26/61) |
| Tracheoesophageal fistula | 57% | (4/7) |
| Intestinal atresia | 38% | (12/32) |
| Cloaca | 25% | (1/4) |
| Cloacal exstrophy | 0% | (0/3) |
| Malrotation | 0% | (0/3) |
| Total | 27% | (97/362) |

Supplementary Table S3: PedsQL scores for patients who were alive, subdivided across digestive tract anomaly (DTA) diagnoses and PedsQL sub-categories. Tracheoesophageal atresia patients scored significantly lower in emotional health and psychosocial health compared to the other DTAs, though overall PedsQL scores remained similar across DTAs. Scores were compared across groups using Student’s t-tests.

|  | Total | Colorectal | Abdominal wall | Intestinal | Esophageal | p-value |
| --- | --- | --- | --- | --- | --- | --- |
| Number of patients | N = 264 | N= 192 | N = 46 | N = 23 | N= 3 |  |
| PedsQL Physical Health | 83.33 (13.96) | 83.43 (13.85) | 82.39 (16.03) | 84.68 (10.58) | 80.67 (15.76) | 0.91 |
| PedsQL Emotional Health | 95.86 (8.83) | 96.58 (8.79) | 93.06 (9.08) | 96.21 (7.75) | 90.28 (8.67) | 0.067 |
| PedsQL Social Functioning | 91.32 (15.27) | 92.74 (13.54) | 85.27 (20.40) | 93.37 (13.52) | 77.50 (19.84) | 0.008 |
| PedsQL Physical Health Summary | 84.60 (12.65) | 84.49 (12.83) | 84.43 (14.24) | 85.74 (7.32) | 85.22 (13.08) | 0.98 |
| PedsQL Psychosocial Health Summary | 93.31 (11.07) | 94.46 (10.04) | 88.17 (13.82) | 95.74 (8.72) | 80.35 (17.32) | <0.001 |
| PedsQL Total Score | 89.71 (10.03) | 90.29 (10.12) | 86.73 (10.60) | 91.72 (6.04) | 82.62 (12.68) | 0.068 |
